# ANN and machine learning based predictions of MRR in AWSJ machining of CFRP composites

**DOI:** 10.1038/s41598-025-92602-8

**Published:** 2025-04-23

**Authors:** K. Ramesha, N. Santhosh, B. A. Praveena, Banakara Nagaraj, N. Channa Keshava Naik, Quadri Noorulhasan Naveed, Ayodele Lasisi, Anteneh Wogasso Wodajo

**Affiliations:** 1 Department of Mechanical and Automobile Engineering, School of Engineering and Technology, CHRIST (Deemed to be University), Bangalore, 560 074 India; 2 Department of Mechanical Engineering, MVJ College of Engineering, Near ITPB, Whitefield, Bangalore, 560 067 India; 3 Department of Mechanical Engineering, Nitte Meenakshi Institute of Technology, Bangalore, India; 4 Department of Mechanical Engineering, Ballari Institute of Technology and Management, Ballari, India; 5 Department of Mechanical Engineering, BGS College of Engineering and Technology, Bangalore, India; 6 Department of Computer Science, College of Computer Science, King Khalid University, Abha, Saudi Arabia; 7 Department of Automotive Engineering, College of Engineering and Technology, Dilla University, Dilla, Ethiopia

**Keywords:** AWSJ, CFRP composites, ANN, ML models, XGBoost, RSM, Engineering, Materials science

## Abstract

This study investigates the effectiveness of Abrasive Water Suspension Jet (AWSJ) Machining, a non-conventional erosion-based method, for machining carbon fiber-reinforced polymer (CFRP) composites. The focus was on analyzing key process parameters—abrasive size, feed rate, and standoff distance (SOD)—under submerged cutting conditions and their impact on material removal rate (MRR), kerf width, and surface roughness. Experimental trials were conducted, and advanced computational techniques, including Response Surface Methodology (RSM), Random Forest (RF), Extreme Gradient Boosting (XGBoost), and Artificial Neural Networks (ANN), were used for parameter optimization and predictive analysis. The results showed that submerged cutting significantly improved machining quality by reducing surface roughness and ensuring uniform kerf widths. Increasing the jet diameter in underwater conditions stabilized the nozzle, leading to smoother and more precise cuts. Among the predictive models, XGBoost demonstrated the highest accuracy and efficiency in forecasting MRR, while Random Forest and ANN provided competitive performance. The integration of RSM and machine learning (ML) techniques enabled effective optimization of machining parameters, showcasing the potential for cost-effective and high-precision CFRP machining. These findings are particularly relevant for industries like aerospace and automotive, where machining efficiency and precision are crucial.

## Introduction

The automotive and aerospace sectors frequently employ carbon-fiber-reinforced plastics because of their exceptional strength and low weight. These materials offer a good mix of stiffness, durability, strength-to-weight ratio, and corrosion resistance^1^. These composites have strong layers that make them challenging to manufacture with conventional methods. This increases tool wear and raises the possibility of splintering and delaminating along the component’s edges. Such defects have the potential to significantly reduce the completed components’ strength^2^.

Abrasive Water Jet (AWJ) machining is the combination of a concentrated, high-pressure water jet with abrasive particles. The sharp edges of these abrasive particles act as cutting edges, causing material to be removed through erosion due to the kinetic energy of the water-abrasive mixture^3^. The non-uniform distribution of abrasive particles in water poses challenges for AWJ machining, despite its advantages, as it diminishes cutting effectiveness.

Abrasive water suspension jet (AWSJ) machining provides an innovative solution to this problem. This method involves increasing the viscosity of water by using a polymer such as Zycoprint. This modification therefore helps to achieve a uniform dispersion of abrasive particles in the water, increasing cutting efficiency for the cutting of CFRP composites. Despite the significance of CFRP composites to the aerospace industry, research on AWSJ for these materials is still in its early stages. Prior studies primarily examine the impact of AWSJ machining process parameters on surface quality, namely surface roughness and kerf width^4–7^.Traditional machining methods fail with CFRP due to rapid tool wear, delamination, poor surface quality, and heat damage. The abrasive nature of CFRP fibers causes excessive wear on tools, while mechanical stresses during machining lead to splintering and delamination of the composite layers. Additionally, conventional methods struggle to achieve smooth surfaces and generate excessive heat, which can degrade the resin matrix and compromise the material’s integrity. AWSJ overcomes these challenges through non-contact, erosion-based machining. It eliminates tool wear by using a high-pressure water jet with abrasive particles, reducing mechanical stresses that cause delamination. Submerged cutting conditions further enhance surface quality by minimizing vibrations and striations, while the water jet dissipates heat effectively, preventing thermal damage and preserving CFRP’s properties.

AWSJ combines high-pressure water with abrasive particles, using polymer additives to enhance performance. The polymer increases water viscosity, ensuring uniform particle suspension and reducing clumping. This improves cutting efficiency by enhancing energy transfer and delivering consistent abrasive impact, resulting in higher MRR, smoother surfaces, and uniform kerf widths. Polymer modification also minimizes air entrapment, stabilizing the jet stream and reducing nozzle wear, making AWSJ highly effective for precision machining of CFRP.

Siva Prasad et al.^8^ carried out several experiments to examine the impact of cutting parameters on surface roughness and kerf taper during the abrasive water jet machining of GFRP composite laminate. The researchers projected surface roughness and kerf taper using mathematical models. The factors that cause striations to appear on the sliced surface were also looked into by the researchers. Manoj et al.^9^ created a statistically generated primary model to investigate the impact of jet angles on the production of high-quality cuts during the multidirectional cutting of aluminium composites using Taguchi DEAR methodology. In addition, an inventive technique for oscillating the cutting head was used to improve the overall quality of cuts during the process. The analysis revealed distinct characteristics in different cut zones. Near the conclusion of the upper zone, when the surface seemed smooth and devoid of pits and striations, the kerf width tapered and reached its minimal width. The kerf width in the intermediate zone was the same as in the upper zone, and despite the absence of pits, striations could be seen. Finally, the lower zone, which was full of pits, showed a discernible ballooning formation and a gradual change in the kerf curvature. Arun et al.^10^ analyzed the cut surfaces in silicon filler based glass fiber reinforced polymer composites using scanning electron microscopy to gain a proper insight into their structures. In this regard, the results have revealed that many parameters can cause the development of striations. These are: wavy abrasive particles, the alterations in kinetic energy distribution, variances in the traverse speed, pressure and abrasive flow rate parameters of the Abrasive Water Jet Machining (AWJM) process and the influence of vibrations in the workpiece and nozzle traverse system. Madival et al.^11^ conducted experiments on the influence of workpiece thickness and feed rate on the deformation effect of varying workpiece thicknesses of rice straw reinforced hybrid polymer composites. It is necessary to analyze the ultimate surface quality for cutting wear processes and deformation wear. Results from the investigation reveal that the mechanism of cutting wear gives better surface quality compared with the mechanism of deformation wear. The research suggested avoiding the cutting of materials that have a thickness of less than certain limit because higher pressure is said to be deleterious on thin materials. Murthy et al.^12^ The experiments were conducted to study the abrasive water jet machining effects on the surface roughness (Ra) and kerf taper ratio (Tr) of jute fibre reinforced polymer composite laminates. An empirical model was developed to study the influence of machining factors on surface roughness and kerf taper ratio. According to their research results, it was concluded that a more rigid abrasive material produced better machining results. They also found a linear relationship between improved machining efficiency and increased hydraulic pressure and abrasive mass flow rate. In contrast, when the speed at which the tool moves and the distance between the tool and the workpiece were decreased, there was a possible improvement in machining process characteristics. Nair et al.^13^ used gray relational analysis in order to improve the.

Dimensions and shape of the hole drilled with a jet. The combined impacts of individual factors on the parameters chosen for experiment were analyzed step by step. The results verify that the standoff distance, the pressure of water jet, and the mass flow rate of the abrasive have been successfully adjusted, and therefore machined surfaces of Inconel 617 demonstrate superior characteristics. Ravi Kumar et al.^14^ successfully applied response surface technology to optimize the process of abrasive water suspension jet machining. This required a thorough analysis of experimental trials and their results, focusing on important details and verifications. They focused their efforts on optimizing the parameters of abrasive jet machining for composites made from aluminum and tungsten carbide. According to the published data, the selected ideal parameters increase the rate of material removal and result in a 22% decrease in surface roughness. The improvement that can be observed is a result of careful selection of the standoff distance and transverse speed at every stage of machining. Miao et al.^15^ found a unique relationship between surface roughness, nozzle diameter, and abrasive grain size when studying the influence of nozzle diameter and abrasive particle size on composite surface roughness using optimization and simulation techniques. This relationship is very important to achieve improved machinability in abrasive water suspension jet machining. Anjaiah et al.^16^ investigated a sequence of experiments aimed at determining the effects of low-pressure abrasive water jets on brittle materials. They established that higher pressures are in direct proportionality to the enhancement of metal removal rates in brittle materials. The researchers also checked the effect of polymer liquid concentration on MRR, where they had observed a proportional positive relationship between percentage polymer in slurry and the MRR. Brandt et al.^17^ were the first who conceived a bypass-based first suspended jet of abrasive water in a configuration that can work at pressure up to 200 MPa. The system has a storage vessel for water, a dense mixture of abradant with polymerized water. To ensure cutting with accuracy of the specified workpiece material, the mixture is passed to the cutting system and delivered as the focused stream to the workpiece. The literature now in publication reveals a common trend of research into abrasive water suspension jet machining: the workpiece is placed above the water. Moreover, optimization of machining parameters has been mainly based on statistical methods other than Taguchi methods^18–20^. Using an abrasive water suspension jet that is “above water” poses a risk since it may entrap air in the jet, expanding it and affecting machining parameters such as surface roughness and kerf width. With these results in mind, the current investigation aims to yield novel results. The aim is to improve the possibilities of work into this field by optimizing process parameters using Taguchi techniques and supporting the process using “Minitab and Design Expert Software”^21–24^. Dahiya et al. reviewed abrasive water jet (AWJ) machining of composites, focusing on its benefits, such as minimal thermal damage and the ability to work with complex materials. They discuss the problems like kerf taper, striation marks, and non-uniform material removal, stating that parameter optimization is required to improve surface quality and accuracy. The paper discusses recent developments in hybrid machining and ML tools, such as ANN and genetic algorithms, for performance optimization. These findings are aligned with the existing work on AWSJ machining of CFRPs to support the applicability of ML for precise prediction of parameters and better machining results^25^. Dahiya et al. carried out further work on the simultaneous optimization of process parameters in AWJ machining of GFRP. The study highlights the significance of interaction effects in parameter optimization for machining performance and the integration of statistical and computational tools in robust optimization. The present research on AWSJ machining of CFRPs is aligned with the study, reinforcing the importance of precise parameter control and optimization to address challenges like kerf irregularities and surface defects. It also supports the application of advanced techniques, such as machine learning, to further enhance machining quality and efficiency in composite materials^26,27^. The difficulties of machining carbon-fiber-reinforced polymers (CFRP) with conventional and traditional abrasive water jet (AWJ) methods—which are hampered by tool wear, delamination, thermal damage, and uneven surface quality—are the focus of the study topic. Current techniques find it difficult to provide the accuracy and effectiveness needed for crucial applications in the automotive and aerospace sectors. Effective methods to improve machining parameters for CFRP are lacking in current research, especially when it comes to resolving problems like uneven material removal, kerf abnormalities, and striations. AWSJ machining is justified by its capacity to get around these restrictions by enhancing the dispersion of abrasive particles, lowering mechanical and thermal stresses, and increasing surface quality and dimensional accuracy. In order to close the discovered gap, this work further incorporates cutting-edge computational tools such as response surface methodology (RSM) and machine learning (ML) to optimize parameters and improve machining performance. Additionally, choosing parameters to introduce optimality into the process is a crucial component of AWSJ machining, and this is achieved through the use of Artificial Neural Networks (ANN) and Machine Learning (ML). The goal of the current effort is to optimize the production process while revolutionizing machining accuracy and efficiency by using AI and ML to estimate Material Removal Rate (MRR) in Abrasive Waterjet (AWSJ) Machining of Carbon Fiber Reinforced Polymer (CFRP) composites. AI/ML techniques are essential in predicting the outcome of AWSJ machining and optimization of parameters. Models such as Random Forest, XGBoost, and ANN are trained on experimental data related to input parameters [abrasive size, Standoff Distance (SoD), concentration, feed rate]. Data preprocessing, training-validation splits, and performance metrics [R^2^, Mean Square Error (MSE), Mean Absolute Error (MAE)] ensure accuracy. AI/ML enables precise predictions, reduces experimental trials, and identifies ideal parameter combinations, significantly improving machining efficiency and control over the traditional counterpart. The aim of this research work is to enhance the machining efficiency of a workpiece material with inherent machining difficulties by predicting the MRR in CFRP, with accuracy. With the utilization of advanced computational techniques like ANN and ML, it aims to make higher precision prediction of MRR in machining and better outcomes from the machining process without wastage of the material, thus improving the manufacturing process with optimal AWSJ machining parameters. This work is novel because it applies new computational methods to challenges inherent in traditional empirical models and thus opens up the possibility of more efficient and optimized CFRP machining processes.

## Experimentation

In the current experiment, MRR, kerf width, and surface roughness are measured in submerged cutting conditions using CFRP as the base material and the AWSJ process applied to it. The main reason why CFRP is chosen is that it is brittle in nature and hence ideal for AWSJ cutting. The following dimensions for the workpiece are required for the completion of AWSJ: 75 mm in length, 50 mm in breadth, and 6 mm in thickness. The CFRP laminates are made using hand layup processes by using EPOXY-ASC resin mixed with HY951 hardener as the matrix phase and carbon fibres as the reinforcements.

The manufacture of the CFRP composite laminate included the subsequent procedures.

The surface is carefully prepared to be free of abrasions and debris and to retain complete flatness. A gel release coat is applied to enable the easy release of CFRP laminates.

The matrix phase is created by mixing the required quantity of epoxy resin with the hardener at a weight ratio of 10:1. Thoroughly agitate the mixture to ensure that the weight ratio of the fiber to the resin and hardener blend is within the permitted range of 40:60.

Furthermore, a resin-hardener combination is applied as a thin layer on top of the release coat. Following this, PAN-based carbon fibers are placed in layers on the surface, and a second significant layer of EPOXY-ASC resin-hardener mixture is evenly spread over the carbon fiber.

Intermittent rolling is done on this layer covered with a thin plastic film sprayed with wax to make removal easier. The rolling operation is carried out with uniform pressure to provide adequate penetration.

Subsequently, 8 more layers of reinforcements and matrix are added one after the other until a laminate with a thickness ranging from 3.9 mm to 4.1 mm is obtained for testing under AWSJ in both free air and underwater cutting scenarios.

To attain the intended surface texture, a fine coat of resin and hardener blend is meticulously administered. The CFRP laminate composite is cured for 24 h and then post-cured in an oven at 120 °C for an additional 5 h.

The CFRP composite work piece is cut to the desired dimensions using the AWSJ method, which is optimized to analyze the impact of process parameters using RSM, AI and ML techniques. Suitable ML algorithms are selected according to the number of input elements and their levels, and are validated throughout the entire experiment to achieve acceptable results with a limited number of trials. The input parameters—abrasive size, standoff distance (SOD), abrasive concentration, and feed rate—are chosen based on their critical influence on the machining process, supported by prior studies and practical relevance in AWSJ machining of CFRP composites. The output parameters—Material Removal Rate (MRR), top kerf width (TKW), back kerf width (BKW), and surface roughness (SR)—were selected to comprehensively evaluate machining efficiency, precision, and surface quality. The choice of a parameter for further optimization depends on the total degree of freedom (DOF), which is derived from the primary effects of all elements in the experiment. The selected process parameters for AWSJ process include abrasive size, standoff distance (SOD), abrasive concentration, and feed. The study examines abrasive sizes of 100 grit, 120 grit, and 140 grit, chosen based on the range of material removed by the abrasive particles. The SOD is adjusted to 1 mm, 3 mm, and 5 mm to accommodate the range of material removal rates observed. Abrasive concentration is adjusted to 100 g, 150 g, and 200 g, while the feed rate of abrasive particles is set at 30 mm/min, 45 mm/min, and 60 mm/min, and a set of nine experimental trials are undertaken after an initial dry run to determine the minimum and maximum ranges for the process parameters. This method, which includes intermittent factors, is effective for accurately determining the overall variable and organizing the results. The 9 experiments are selected using a systematic DOE approach to evaluate key parameters (abrasive size, SOD, abrasive concentration, and feed rate) at three levels each. This ensures efficient analysis of interactions, practical range validation, and optimization of machining outcomes for CFRP composites. The experimental runs were designed with a Box-Behnken Design (BBD) within the Response Surface Methodology (RSM), varying key input parameters such as abrasive size, SOD, concentration, and feed rate at three levels, thereby providing 9 experimental runs. The experimental runs were divided into training and testing datasets; the training data were used to develop machine learning (ML) models, including Random Forest, XGBoost, and ANN, while the testing data were used to validate the predictive accuracy of these models.

RSM initially identified the significant factors and interactions influencing output variables such as MRR, TKW, BKW, and SR, providing response surfaces to visualize these relationships. ML models further optimized the process through desirability-based analysis, predicting optimal parameter combinations to enhance machining outcomes. Validation of the ML models was performed by comparing predicted results with experimental data, with success defined by high predictive accuracy (R^2^ > 0.97) and minimal deviation between predicted and actual values. Effective optimization was demonstrated by achieving higher MRR, lower SR, and overall improved machining efficiency compared to unoptimized conditions or prior studies. This structured approach ensured robust validation of the ML models and the effectiveness of the AWSJ optimization process.

### Cutting parameters

There are three different groups of process parameters, which are the abrasive suspension parameters, nozzle characteristics, and system operational parameters, which affect the cutting process. The process is very much influenced by these factors. A critical analysis of the parameters has revealed that there is a complete understanding of the specific factors for the proper modeling of the process. This knowledge gave rise to certain criteria for pilot tests.

The experiments are so conducted that suspensions by an underwater jet with typical atmospheric circumstances are created after careful consideration, with utmost concern for ambient pressure of air in the vicinity 101.325 kPa, air density = 1.225 kg/m^3^ and, temperature T at 288.15 K, which constitute regulation of strong elements for establishing highly reliable repeatable experimental protocol assuring detailed observation of all intrinsic processes involved.

### Measurement of output parameters

To guarantee accuracy and dependability, precision equipment was used to measure the output characteristics. A Shimadzu AUW220 analytical balance with a capacity of 220 g and an accuracy of ± 0.01 g was used to measure the weight difference of the workpiece before and after machining in order to compute the Material Removal Rate (MRR). A Leica DM750 optical microscope with a 50x–500x magnification range and a measurement precision of ± 0.01 mm was used to measure the Top Kerf Width (TKW) and Back Kerf Width (BKW), guaranteeing accurate kerf dimension profiling. Surface Roughness (SR) was measured by a Mitutoyo Surftest SJ-410 surface profilometer, supplied with a 2 μm stylus tip, a range of ± 200 μm, and an accuracy of ± 0.02 μm. These high-resolution measuring instruments assured the accuracy of the data regarding the machining outcomes.

## Application of soft computing to optimize the parameters

Response Surface Methodology (RSM) is used to consider the effects of input parameters: Abrasive size, Stand-off distance (SOD), feed rate, Abrasive concentration on the output responses material removal rate, top kerf width, back kerf width, and surface roughness. The parameters involved are varied at three levels as per the BBD, while considering the interaction effects and other non-linear relationship in the outputs.

ANOVA has been performed in order to study the significant parameters and their interaction. F-value and p-values less than 0.05 have been determined for the importance of the parameter. R^2^ and Adjusted R^2^ have estimated the overall fitting and predictability of the model. SOD was found out to be more significant in influence on MRR and surface quality. The results of the developed model were cross-validated through MSE and MAE. Further, contour plots have been produced to visualize the interaction between parameters.

Model validation was based on the comparison of predicted results with experimental outcomes, which is further justified using metrics such as Predicted R^2^ and Adeq Precision. RSM findings were then complemented using machine learning models: Random Forest, XGBoost, and ANN, which give highly accurate predictions and optimize parameters to the optimal level. A combination of both RSM and ML techniques, therefore, would ensure precise modeling and robust optimization of AWSJ machining outcomes.

### Response surface methodology

The response surface model (RSM) has been used in establishing the response surface model and stressing effects of input parameters and target functions on interactions and linkages among these components. The response surface model may be expressed in numerous manners, including the following:

1 $$y=f\left(D,P,\partial\right)+\gamma$$

In this equation, $y$ represents the intended response, while $\gamma$ stands for the response error. When applying a parametric model to an objective function, it is customary to utilize a second-order response surface model. Here is a potential depiction of the model:

2 $$y={\beta}_{0}+{\sum}_{i=1}^{n}{\beta}_{i}{x}_{i}+{\sum}_{i=1}^{n}{\beta}_{ii}{{x}_{i}}^{2}+{\sum}_{i=1}^{n}{{\sum}_{j>1}^{n}x}_{i}{x}_{j}+\gamma$$

In Eq. (2) ${\beta}_{0}$denotes a constant term, ${\beta}_{i},{\beta}_{ii},and{\beta}_{ij}$are regressions terms coefficient of respective order. Herein, n denotes count of terms while x being the independent variable.

#### Box-Behnken design

A statistical experimental design used in RSM is called the Box-Behnken design (BBD). It is feasible to reduce the number of experimental runs required without sacrificing the ability to examine the ways in which various factors influence a process’s response. In order to complete the design, a precise set of component values falling within predetermined ranges must be obtained. Usually, each element consists of three levels, with the middle level lying in between the high and low levels. This approach makes it possible to analyze the effects of variables on the result in both quadratic and linear forms. A BBD’s goal is orthogonality, which guarantees that the effects of its components are as independent as is practical. This feature makes it possible to estimate the response surface model’s coefficients with greater accuracy. It can be characterized as follows:

The variable “i” represents the degree of the “i” th factor, with “i” ranging from 1 to “k”, where “k” is the total number of factors. The BBD design chooses n combinations of factor levels denoted as xi1, xi2, xi3… xik, where i ranges from 1 to n. Each set of factor values is linked to an estimated response variable (yi). The dataset serves as a design matrix for subsequent study through analysis of variance (ANOVA).

The number of experimental runs required by a BBD depends on the factors being investigated. A regression model is fitted to the data after conducting tests and collecting data on the response variable to quantify the influence of the variables and their interactions on the result. This model demonstrates the relationships between variables and responses using mathematical expressions. The BBD provides several benefits. Reducing the number of necessary experimental runs leads to cost and time savings. They demonstrate efficiency in this area. In addition, they streamline the implementation process compared to other experimental designs. These setups allow for evaluating both the linear and quadratic impacts of the components, leading to a comprehensive understanding of the underlying mechanism. BBDis widely used in industries including pharmaceuticals, chemistry, and engineering for optimizing processes and products. By pinpointing the optimal operating parameters and comprehending the relationships between influencing aspects, they significantly enhance efforts to boost productivity, quality, and execution.

#### Desirability-based optimization

The Desirability-based optimization technique is commonly employed to enhance multiple response procedures. Numerical optimization procedure identifies the point where the desirability function reaches its maximum value. The method identifies and describes the operating circumstances that yield the most optimal response values. The prime factors and the corresponding reaction are explicitly defined by ramp function graphs. Numerical optimizations were used to find the mix of parameters that would yield the most favorable outcome to achieve the targeted objective. Each ramp is color-coded to show the factor setting or response prediction of the solution. Gray dots on the ramp function graph reflect optimal response prediction values, whereas red dots show optimum factor settings. Desirability can be quantified by a numerical number ranging from zero to one. It is widely accepted that the number closest to 1 is the most preferable. The quadratic model was utilized in this inquiry to create the optimization plot, aiding in predicting the answer with a favorable outcome.

### Machine learning

The process of machine using this unconventional machining method shows complex and non-linear behaviour. The data thus generated is difficult to model-predict using conventional methods and numerical analysis. Modern machine learning methods can help in this regard. Hence, in the present study, two such ML approaches Random Forest (RF) and eXtreme Gradient Boosting (XGBoost) were employed in the present study.

#### Random forest

The random forest (RF) method is called as ensembling approach, and it entails the development of many decision trees to anticipate. It can support a large number of input variables without the necessity for variable selection, and it does not overfit the dataset. It may be used to build regression and classification models. This inquiry yields two random forest regression models, one for each output parameter, by creating a link between the machining process control factors and its output process parameters. The bagging strategy, also known as the bootstrap aggregation technique, is used to reduce the incidence of overfitting and excessive variation in decision tree algorithms. During the bootstrap phase, multiple samples are extracted from the original training dataset using replacement. This means that selected records from one sample may be replicated in another sample. The decision tree constructed for each unique sample results in the formation of a random forest or a random decision tree forest.

In RF method the forecasted output of $\widehat{y}$ a given control factor x using mean prediction of multiple decision trees from a group of trees termed as forest. In this case, each tree predicts an independent$\widehat{y}$, and then the final prediction is estimated by averaging all predictions.

Suppose the total number of trees in the forest is ‘n’ and the forecast from the i_th_ decision tree is ${\widehat{y}}_{i}\left(x\right)$, in such case the overall forecast can be described as:

3 $$\widehat{y}\left(x\right)=\frac{1}{n}{\sum}_{i-1}^{n}{\widehat{y}}_{i}\left(x\right)$$

In practice, each decision tree is trained using a bootstrap sampling of the original dataset, with a random subset of characteristics considered at each split. This unpredictability aids in de-correlating the trees and prevents overfitting.

In addition, random forest regression might include weights for each tree to account for their relative relevance in formulating predictions. Let ${w}_{i}$ represent the weight applied to the i_th_ tree. The weighted average forecast may be expressed as:

4 $$\widehat{y}\left(x\right)=\frac{1}{{\sum}_{i-1}^{n}{w}_{i}}{\sum}_{i-1}^{n}{{w}_{i}\widehat{y}}_{i}\left(x\right)$$

The average output of the many tree models is employed in the aggregation step of the random forest regression algorithm. In contrast, in random forest classification, the tree with the most votes from all of the trees determines the final decision. Changing the hyper parameter settings for the random forest approach may increase the algorithm’s prediction accuracy.

#### XGBoost

Extreme Gradient Boosting, or XG Boost, is a widely employed, popular, and robust machine learning approach that is well-known for its efficacy and ease in manipulating tabular data. It is a member of the ensemble learning technique family, and it works by iteratively assembling an ensemble of weak learners, commonly decision trees, to produce a strong prediction model. XG Boost performs well in both classification as well as regression because it uses regularization to reduce overfitting and minimizes a specific loss function. Two of its primary qualities are gradient boosting, which corrects prediction errors produced by previous models sequentially, and approximation tree learning, which speeds training by building trees level-wise. Both of these features are among its most crucial. XG Boost’s success may be due to its ability to create highly accurate predictions as well as its versatility in a wide range of applications. As a consequence, it is rapidly becoming the favored tool among data scientists and machine learning practitioners. The process was as follows:

In case the used training data set is specified as-

5 $$T={\left\{\left({x}_{i},{y}_{i}\right)\right\}}_{i=1}^{n}$$

Herein, the ${x}_{i}$ is the feature vector while ${y}_{i}$represents the label for i_th_ sample. XGBoost ML aims to train the additive ensemble of weak learners:

$F\left(x\right)={\sum}_{k=1}^{K}{f}_{k}\left(x\right)$ ; in this case each ${f}_{k}\left(x\right)$ represents a regression tree.

The objective function of XGBoost is denoted as:

6 $$Obj = \mathop \sum \limits_{{i = 1}}^{n} L \;(y_{i} , \; {\overbrace {y}}_{i} ) + \mathop \sum \limits_{{k = 1}}^{K} {{\Omega }}\left( {f_{k} } \right)$$

Whereas$L({y}_{i}, {\overbrace {y}}_{i})$ denotes the loss function ${y}_{i}$ is the measure value and ${\overbrace {y}}_{i}$ represents the forecasted value.

The model undergoes training repeatedly by adding weak learners to the ensemble. In each cycle, a fresh regression tree is trained to minimize the following objective function:

7 $${Obj}_{k}={\sum}_{i=1}^{n}L({y}_{i}, {\overbrace {y}}_{i}^{t})+{\sum}_{i=1}^{t}{\Omega}\left({f}_{i}\right)+\gamma.T+\frac{1}{2}\lambda{\sum}_{j=1}^{T}{\omega}_{j}^{2}$$

In the above expression, T represents the number of leaves $\gamma$ and $\lambda$ denotes the regularization term defines the tree’s complexity.

Following training, the final forecast for a new sample x is derived by merging the predictions from each regression tree in the ensemble.

8 $$F\left(x\right)={\sum}_{k=1}^{K}{f}_{k}\left(x\right)$$

Whereas the ${f}_{k}\left(x\right)$ is the forecast of k_th_ tree.

#### ANN

**Fig. 1:**
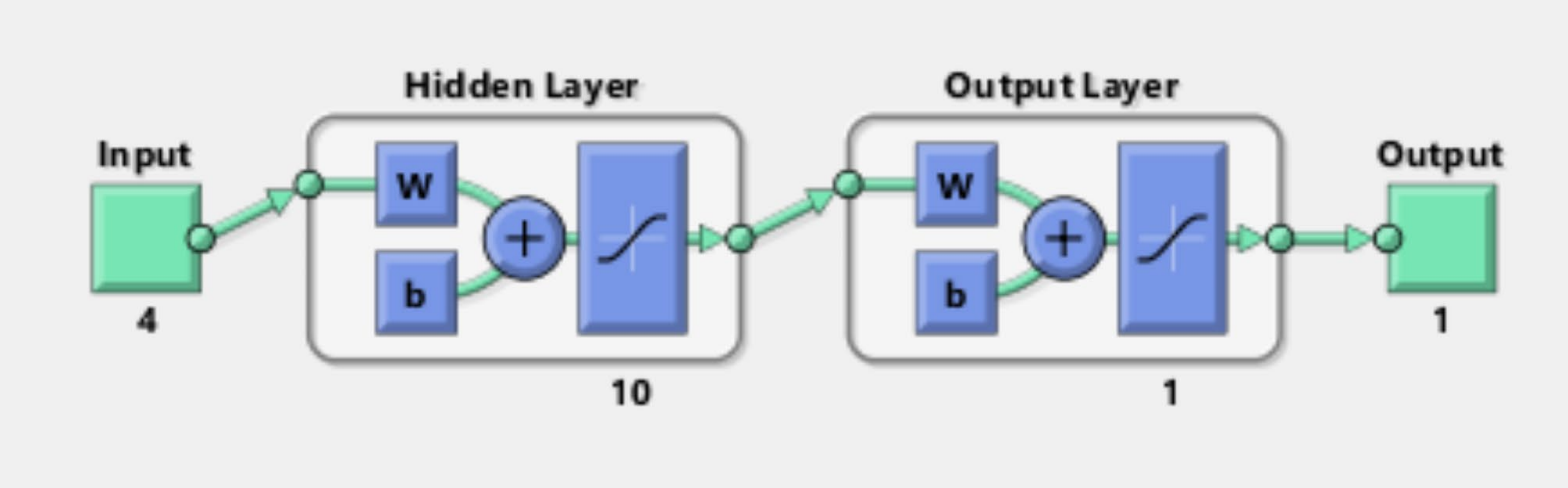
ANN model used in the present work.

Artificial neural networks (ANNs), which are made up of multiple simple processing units called neurons stacked in three layers—input, output, and hidden layers—represent modeling approaches for artificial intelligence. They resemble human brain networks’ interconnected structure quite closely. The Fig. 1 gives the ANN network used in the present work.

An important benefit of artificial neural networks (ANNs) is its capacity for supervised or unsupervised learning using supplied datasets, or “training sets.” To achieve desired results, weights are calculated using learning approaches once the network structure has been built. Artificial Neural Networks (ANNs) come in a variety of topologies, architectures, and training techniques. They can adjust their parameters iteratively based on how well they operate, utilizing the ideas of gradient descent theory.

Since the sigmoid function can behave almost linearly, curvilinearly, or constantly depending on the input values, it is a frequently utilized activation function in artificial neural networks. This function is commonly known as a squashing function because it is bounded between 0 and 1.

As an example of supervised learning, backpropagation neural networks require large datasets that include target variables. Errors are computed by comparing the output values that output nodes provide as each observation moves through the network to the actual target variables. Separating the data into training and testing sets is necessary to assess the performance of the ANN model. Network parameters or synaptic weights are determined using the training set, and learning ends when error objectives are reached. The data from the testing set is then used to test the network. In the present work, the Levenberg-Marquardt training set is used, with the Eq. 9 used for predicting the outcome of the values.

9 $$Z = W_{0}^{^\circ } + W_{1} X_{1}^{^\circ } + W_{2} X_{2}^{^\circ } + \ldots + W_{n} X_{n}$$

Where, Z is the symbol for denotation of the above graphical representation of ANN. W_i_ are the weights or the beta coefficients. X is, are the independent variables or the inputs, and W_0_ represents Bias or intercept

#### Comparison of outcomes of different models

The statistical methods as well as pictorial method of Taylor’s diagram were employed in the present study. R^2^, referred to as the coefficient of determination, measures the extent to which of the variation in the dependent variable (the variable being predicted) is explained by the differences between the independent variables (the predictors) in the regression model. It varies between 0 and 1, where the value ‘1’ perfectly explain the model while the value ‘0’ do not explain at all. The preferred value should be between 0 and 1, but it should be close to 1. It is expressed as:

10 $${R}^{2}=1-\frac{{SS}_{res}}{{SS}_{tot}}$$

Herein, ${SS}_{res}$ denotes residual’s sum of squares while as ${SS}_{tot}$ total sum of squares.

Mean Squared Error calculates the average squared difference between actual and anticipated values. It measures the average size of the model’s mistakes, with higher numbers representing bigger faults. The MSE is computed as:

11 $$MSE=\frac{1}{n}{\sum}_{i-1}^{n}{({y}_{i}-\widehat{{y}_{i}})}^{2}$$

Wherein, n is the number of observations, ${y}_{i}$ denotes the actual value of i_th_ observation, $\widehat{{y}_{i}}$ represents the predicted value of i_th_ observation.

Mean Absolute Error calculates the average absolute difference between actual and anticipated data. It offers a measure of the average size of the model’s mistakes, similar to MSE, but it penalizes huge errors less severely. MAE is computed as follows:

12 $$MAE=\frac{1}{n}{\sum}_{i-1}^{n}\left|{y}_{i}-\widehat{{y}_{i}}\right|$$

## Results and discussion

### Experimental results and discussion

Table 1 represents the underwater cutting experimental setup and results, where different factor values have been evaluated to study the effect on different reaction characteristics. Numbered from 1 to 9, every experiment tests a different combination of abrasive size, feed rate, stand-off distance (SOD), and abrasive concentration. It then measures the effect of these combinations on surface roughness (SR), material removal rate (MRR), and top and bottom kerf widths (TKW and BKW, respectivelyThe researchers observe differences in the breadth of cut at various depths, smoothness of the surface machined, and effectiveness of the material removal based on variations in parameters such as abrasive size, stand-off distance, concentration, and feed rate. To enhance the efficiency and quality of underwater cutting applications across various industries, the data indicates that ideal conditions for these processes include striving for higher material removal rates, more accurate kerf widths, and better surface finishes. The experimental setup and parameter selection were designed with careful structure for robustness and reproducibility of results. Parameters, including abrasive size, standoff distance (SOD), abrasive concentration, and feed rate, were varied systematically using a Box-Behnken Design (BBD) under Response Surface Methodology (RSM). This will enable the study of three levels for each parameter, such as grit sizes 100, 120, 140; SOD 1 mm, 3 mm, 5 mm, and minimize the number of trials required for statistical significance. Parameter levels used were chosen based on their influence on machining outcomes, supported by material characteristics of CFRP and prior research findings. Abrasive size levels selected would balance material removal rate and surface quality, while ranges for SOD, abrasive concentration, and feed rate were optimized to evaluate the combined effects on cutting performance, kerf width, and surface roughness. The experimental trials were randomized to reduce bias and ensure statistical validity. Although each experiment was performed only once, RSM ensures that modeling and interaction analysis are robust. This design will thus support reproducibility and reliability of results, capturing interactions between factors and reducing experimental effort.

**Table 1:** Factor settings and response parameters for AWSJ.

Expt. no.	Abr. Size (grit)	SOD (mm)	Abr. con. (g)	Feed (mm/min)	MRR (g/min)	TKW (mm)	BKW (mm)	SR (µ)
1	#100	1	100	30	2.14	1.79	0.80	8.16
2	#100	3	150	45	2.57	1.83	0.84	8.73
3	#100	5	200	60	2.89	1.78	0.78	8.85
4	#120	1	150	60	2.51	1.56	0.71	8.96
5	#120	3	200	30	2.98	1.82	0.84	8.22
6	#120	5	100	45	3.34	1.88	0.79	8.76
7	#140	1	200	30	2.65	1.70	0.81	8.69
8	#140	3	100	60	3.11	1.54	0.67	9.1
9	#140	5	150	45	3.64	1.85	0.84	8.21

The recorded response data in Table 1, including Material Removal Rate (MRR), Top Kerf Width (TKW), Back Kerf Width (BKW), and Surface Roughness (SR), have undergone critical analysis, and the MRR which is having impact on all the other responses is subjected to RSM, AI and ML based modeling to determine the significant factors influencing the outcomes. An investigation on the analysis of the role of input variables such as abrasion size, feed rate, SOD, and concentration on response variables in the form of MRR, SR, TKW, BKW has been made with statistical analysis using RSM based Analysis of Variance (ANOVA). In these studies, it has been considered to assess statistical measures, the effect of interactions of individual inputs under consideration using their F-values and respective p-values, thereby defining significant measures of responsesSignificant factors are identified based on p-values below 0.05, which indicates a strong influence on response parameters. Interaction effects, such as abrasive size and feed rate, and quadratic effects, such as SOD^2^, are also analyzed to capture non-linear relationships. Fit statistics, including the coefficient of determination (R^2^), adjusted R^2^, and predicted R^2^, ensured the accuracy and reliability of the model. These statistical methods validated the experimental findings and provided robust support for the conclusions drawn regarding the influence of process parameters on AWSJ machining outcomes.

### Data pre-processing and correlational analysis

**Fig. 2:**
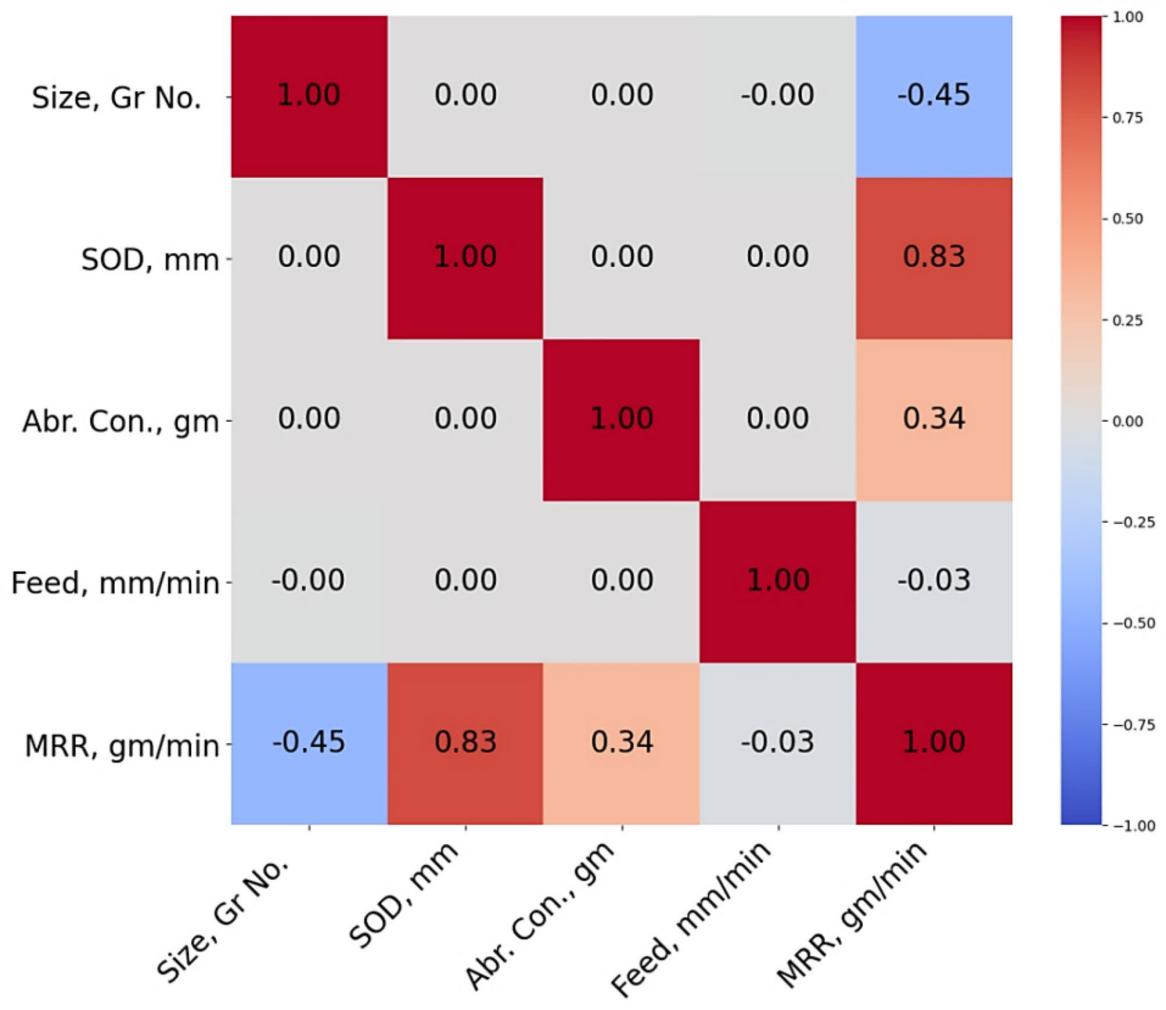
Correlational heatmap.

Data preparation, which involves creating correlation heatmaps, ensures the analytical integrity, trustworthiness, and efficacy. These procedures are important in research and analysis for the following reasons. Correlation heatmaps depict the relationships between dataset variables. They let researchers to assess how variables interact, whether they are favorably or negatively connected, and to what extent. This information is critical for selecting model predictors and spotting potential multicollinearity issues. Identifying strong links can help you pick features by eliminating duplicate data points that are irrelevant to the inquiry. Figure 2 shows a correlation heatmap for the research data.

**Table 2:** Correlation values.

	Size, Gr No.	SOD, mm	Abr. Con., g	Feed, mm/min	MRR, g/min
Size, Gr No.	1	0	0	0	-0.4505
SOD, mm	0	1	0	0	0.8270
Abr. Con., g	0	0	1	0	0.3354
Feed, mm/min	0	0	0	1	-0.0264
MRR, g/min	-0.4505	0.8270	0.3354	-0.0264	1.0000

The presented Table 2 shows the correlation matrix, with each row and column representing a separate variable or parameter. The correlation coefficient “1” along the diagonal implies perfect correlation with itself, as expected. Similarly, the second row and column indicate the variable “SOD, mm”. Again, a correlation coefficient of “1” across the diagonal shows perfect correlation with itself. Each cell in the table represents the correlation coefficient of the associated pair of variables. As an illustration, the correlation coefficient of “-0.4505” is observed in the cell where the row is labeled “Size, Gr No.” and the column is labeled “MRR, gm/min”. This coefficient indicates a negative association between the size/grade number and the material removal rate. The correlation coefficient between the cell in the “Feed, mm/min” row and the “SOD, mm” column is “0.8270”. It shows a positive correlation between feed rate and depth of cut. In general, the correlation matrix provides insights into the interrelation among variables in the dataset. It aids in understanding the relationship between variables and is fundamental to data analysis and informed decisions in fields like manufacturing, engineering, and research.

#### ANOVA (analysis of variance)

The Table 3 (ANOVA table) reports the results of a factorial experiment that evaluates the effects of four factors, A-Size, B-SOD, C-Abrasive Concentration, and D-Feed, and their interactions on a response variable. As shown by the statistical significance of the model at p-value < 0.0001, at least one of the factors or interactions impacts the response variable. The p-values (< 0.0001) demonstrate that Factor A (A-Size), Factor B (B-SOD), Factor C (C-Abrasive Concentration), and Factor D (D-Feed) all significantly affect the response variable. Additionally, the response variable is significantly impacted by a number of interactions between the components (AB, AC, AD, BC, and CD), suggesting that these factors’ combined influence goes beyond simple additive effects.

Furthermore, the presence of significant quadratic effects (A^2^, B^2^, C^2^, D^2^) indicates that the relationship between some factors and the response variable is not strictly linear.

**Table 3:** ANOVA table.

Source	SS	df	Mean square	F-value	*p*-value	
Model	2	14	0.143	48047.33	< 0.0001	Significant
A-size	0.3997	1	0.3997	1.34E + 05	< 0.0001	
B-SOD	1.37	1	1.37	4.62E + 05	< 0.0001	
C-abr. con.	0.2269	1	0.2269	76,230	< 0.0001	
D-Feed	0.0016	1	0.0016	548.8	< 0.0001	
AB	0	1	0	8.4	0.0117	
AC	0	1	0	8.4	0.0117	
AD	0	1	0	8.4	0.0117	
BC	0	1	0	8.4	0.0117	
BD	0	1	0	0	1	
CD	0	1	0	8.4	0.0117	
A^2^	0	1	0	6.05	0.0275	
B^2^	1.00E-07	1	1.00E-06	0.3784	0.5483	
C^2^	0	1	0	6.05	0.0275	
D^2^	1.13E-06	1	1.13E-06	0.3784	0.5483	
Residual	0	14	2.98E-06			
Lack of fit	0	10	4.17E-06			
Pure error	0	4	0			
Cor total	2	28				

Given that the model has an F-value of 48047.33, it can be concluded that the model is significant. It is extremely unlikely that noise could be the cause of an F-value of this magnitude; the probability is only 0.01%. P-values that are lower than 0.0500 imply that the model terms are statistically significant. There are a number of relevant model terms in this scenario, including A, B, C, D, AB, AC, AD, BC, CD, A^2^, and C^2^. When the values are more than 0.1000, it indicates that the model terms do not have any significance. It is possible that model reduction will improve your model if it has a large number of inconsequential model terms (taking into account just those that are necessary to support hierarchy).

13 $$\begin{aligned} MRR = \; & 2.78 - 0.0111*Sz + 0.157*SOD + 0.002*AC - 0.0024*F + 0.0000625*Sz*SOD \\ & + 0.0000025*Sz*AC + 0.0000083*Sz*F + 0.000025*SOD*AC \\ & - 0.0000033*AC*F + 0.0000042*Sz^{2} + 0.000104*SOD^{2} \\ & + 0.00000067*AC^{2} + 0.0000018*F^{2} \\ \end{aligned}$$

Where Sz represents the size of the abrasive, SOD represents the stand off distance, AC represents the Abrasive Concentration, and F represents the feed.

**Fig. 3:**
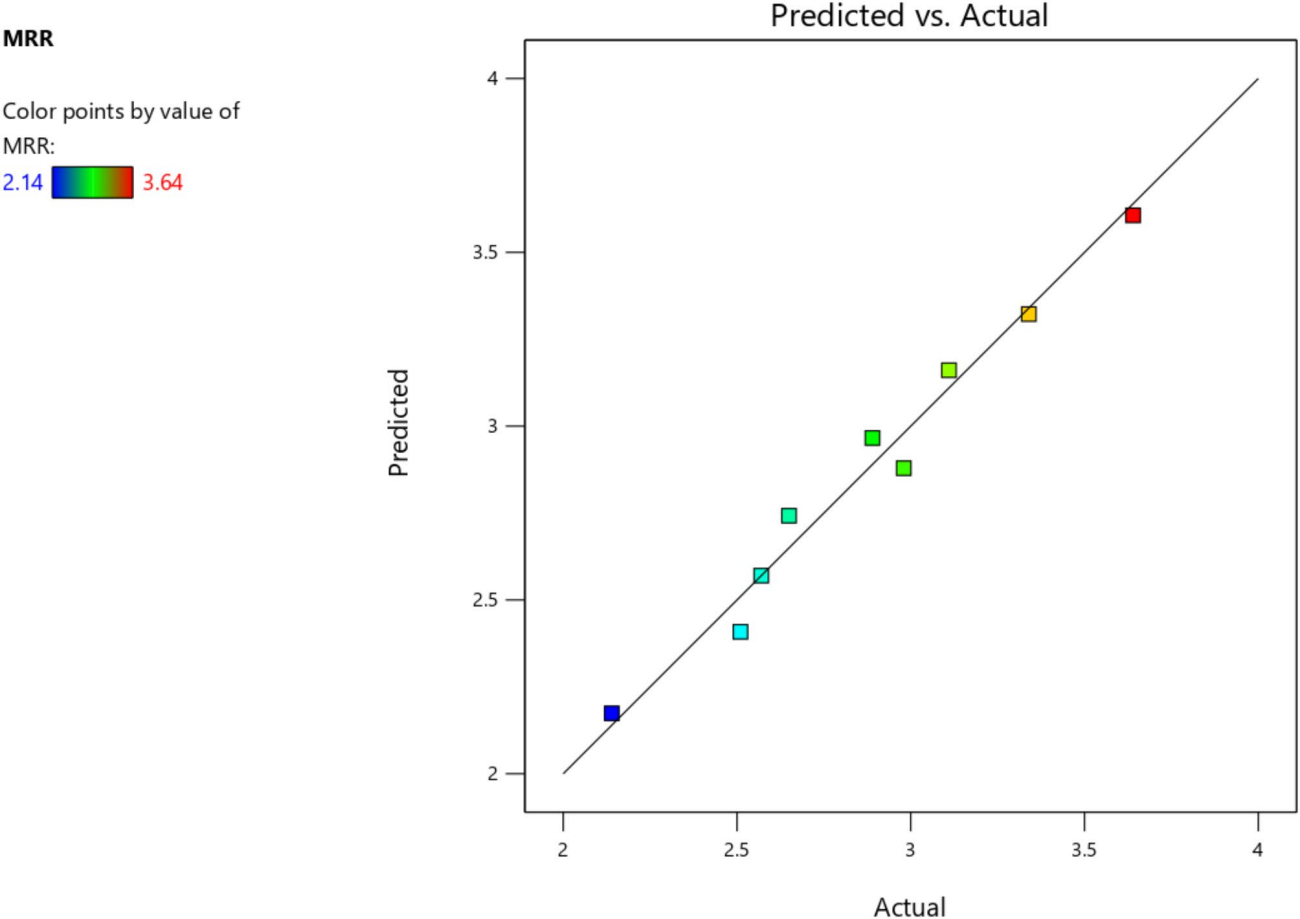
Model predicted vs. actual MRR values.

**Fig. 4:**
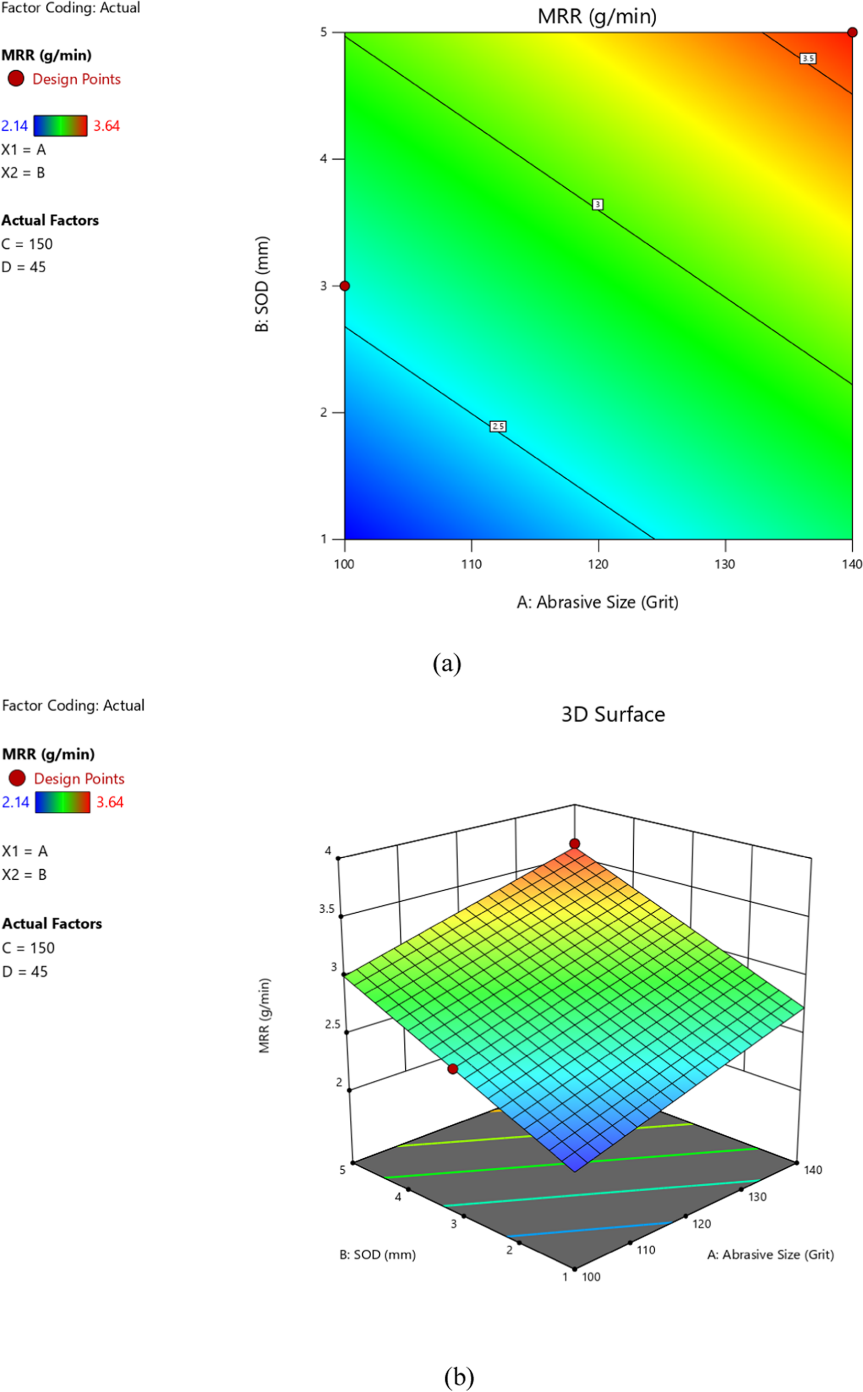

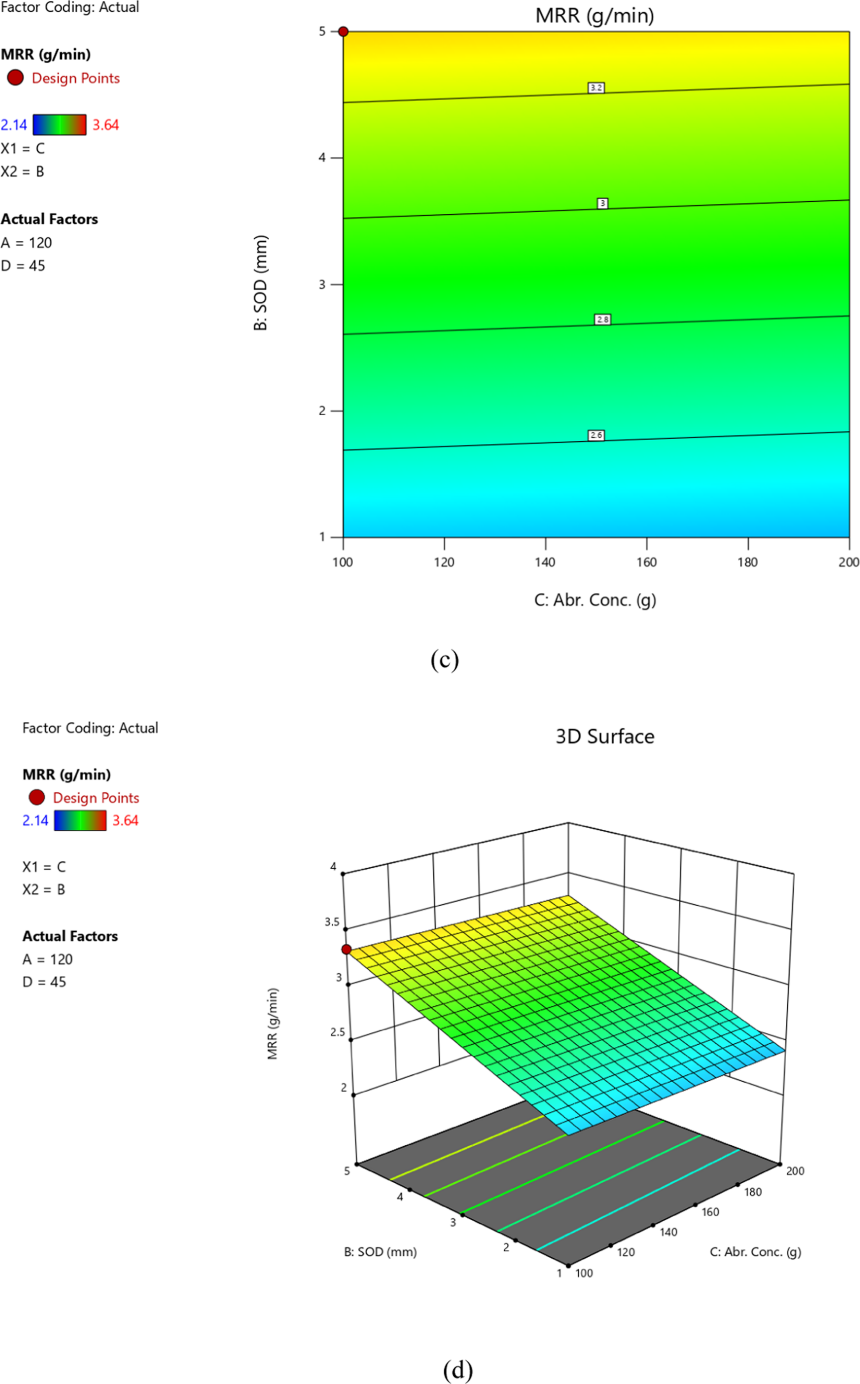

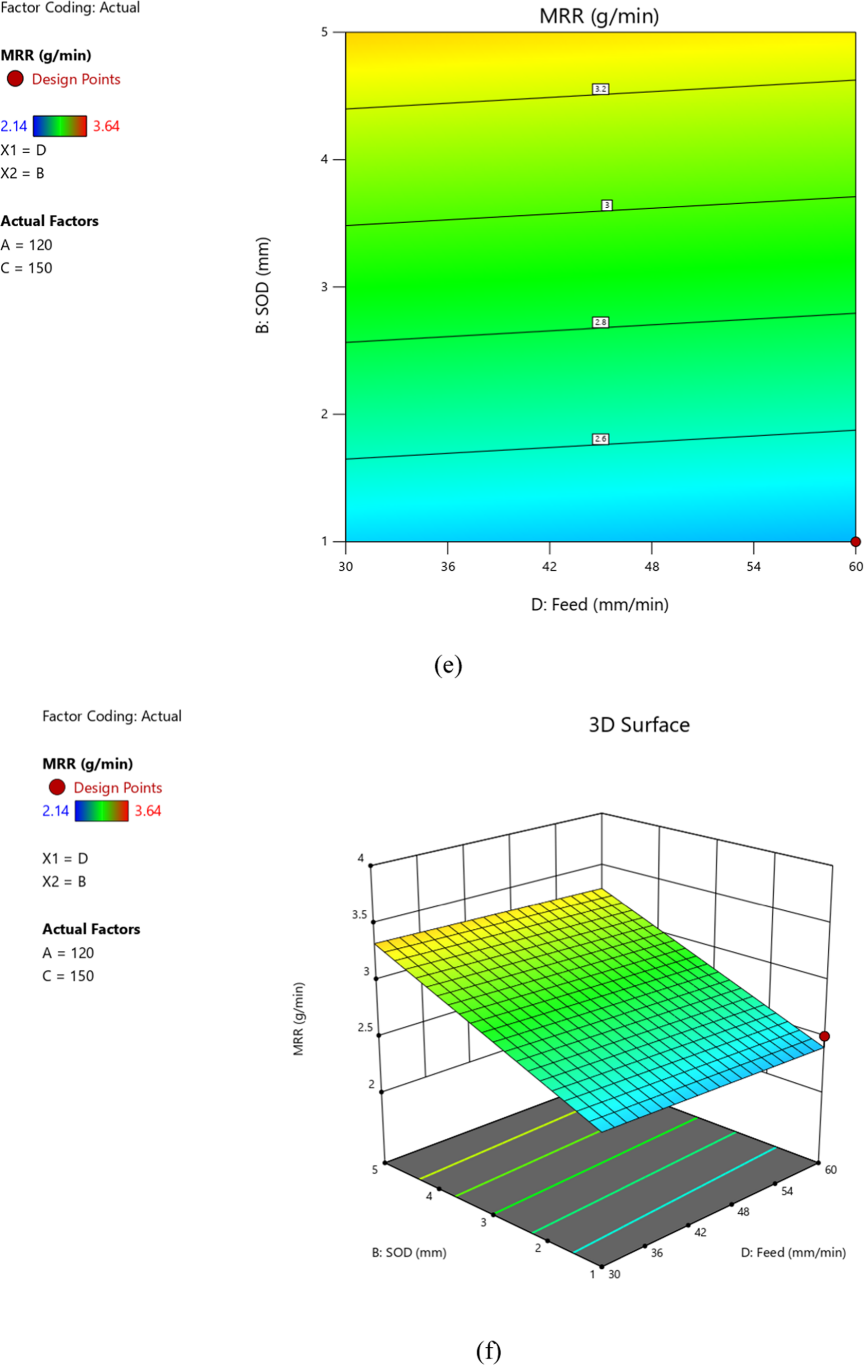
Contour graphs and surface diagrams.

The model predicted vs. actual MRR values are depicted in Fig. 3 as predicted by the model given by Eq. 13. Figure 4 illustrates the relationships between input parameters (abrasive size, standoff distance, feed rate, abrasive concentration) and output responses (MRR, SR, TKW, BKW) through contour graphs and surface diagrams. These visualizations highlight how parameter interactions influence machining outcomes. Graphs (a) and (b) show the impact of standoff distance (SOD) and abrasive size on MRR, revealing that lower SOD and finer abrasive size enhance MRR by improving jet focus and material erosion. Graphs (c) and (d) depict the effects of feed rate and abrasive concentration on surface roughness (SR), demonstrating that lower feed rates and moderate abrasive concentrations result in smoother surfaces. Graphs (e) and (f) illustrate the interaction of feed rate and SOD on kerf width (TKW/BKW), showing that optimized SOD and lower feed rates achieve uniform and narrow kerf widths. These graphs provide valuable insights for optimizing AWSJ machining parameters, enabling improved precision and efficiency in machining CFRP composites.

**Table 4:** Fit statistics from RSM.

Std. Dev.	0.1000	*R* ^2^	0.9763
Mean	2.87	Adjusted R^2^	0.9525
C.V. %	3.48	Predicted R^2^	0.8208
		Adeq Precision	19.2251

The Table 4 gives the fit statistics from RSM. The Predicted R^2^ of 0.8208 is in reasonable agreement with the Adjusted R^2^ of 0.9525; i.e. the difference is less than 0.2.

The Adeq precision value > 4 is preferable and the ratio of 19.2251 indicates an adequate signal. Thus, the present model developed from the RSM techniques using Design Expert can be effectively used to evolve the design space for optimization and prediction of input parameters.

### Desirability based optimization and point prediction

**Fig. 5:**
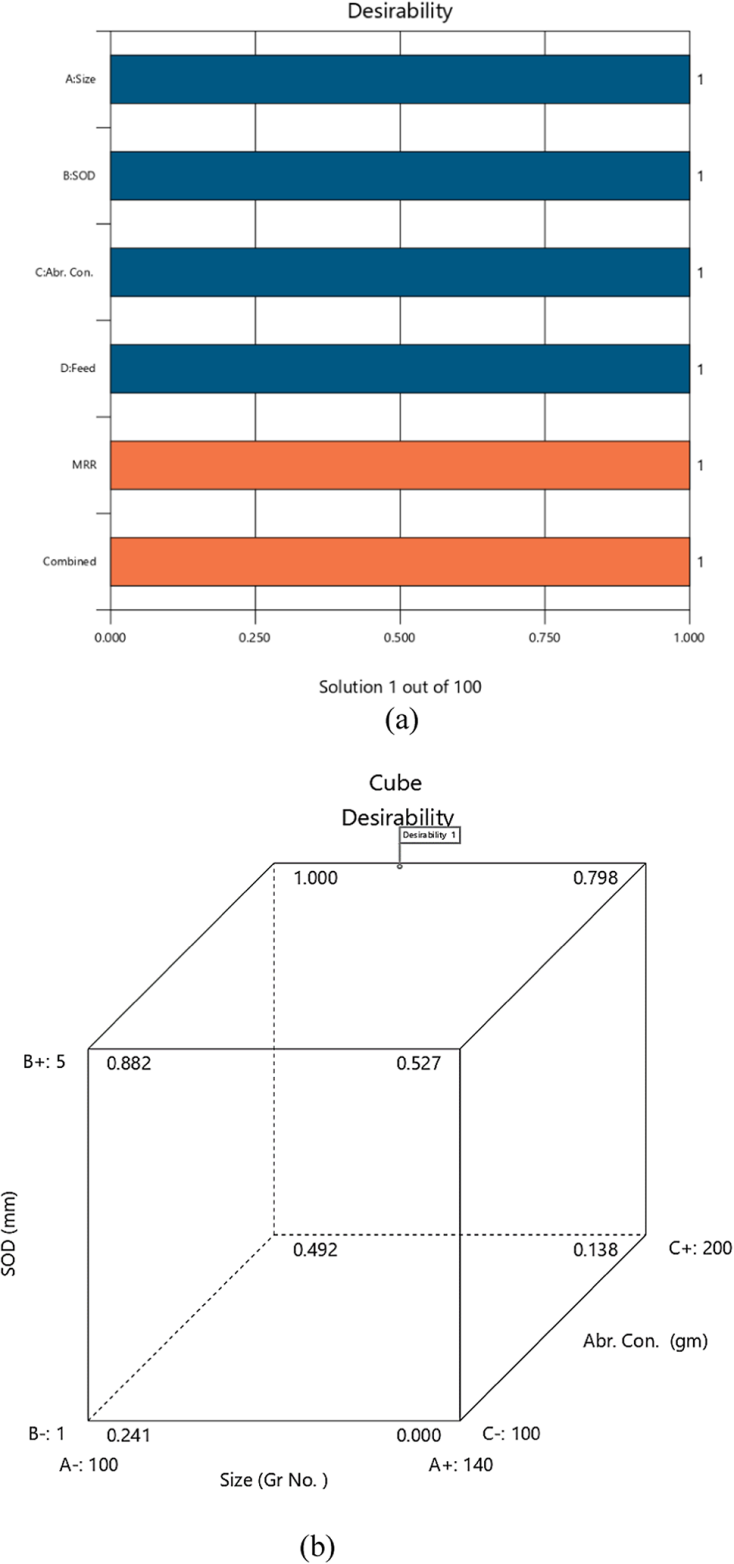
(**a**) Desirability bar. (**b**) Desirability cube.

Desirability-based optimization and point prediction are techniques used in experimental design and analysis to identify the optimal combination of factor levels that maximize or minimize a response variable while considering multiple constraints or objectives. Desirability-based optimization involves assigning desirability values to each factor level combination based on the researcher’s objectives. These desirability values can reflect the importance of achieving specific target values or ranges for the response variable. The desirability function allows researchers to balance multiple objectives and constraints simultaneously. Figure 5 (a) gives the desirability bar, while the Fig. 5(b) gives the desirability cube, which essentially represents Desirability based optimization.

Point prediction, on the other hand, is a statistical technique used to predict the response variable’s value at a specific combination of factor levels. It utilizes regression models developed from experimental data to estimate the response variable’s value under different conditions.

Researchers would first define their optimization criteria and aims for the response variable before applying desirability-based optimization and point prediction in this situation. They would next create models that link the response variable to the various components and their values using statistical techniques like response surface methodology or regression analysis. Finally, they would use these models to identify the optimal combination of factor levels that maximizes the desirability of the response variable while meeting any constraints or objectives set forth by the research study.

From the desirability-based optimization Table 5, the following inferences are drawn.

Factor A (Size) represents the size of a parameter being studied, with a low level of 100.00 and a high level of 140.00, measured in a specific unit (presumably a measurement scale related to the experiment).

Factor B (SOD) refers to another parameter, possibly solution or sample dilution, with a low level of 1.0000 and a high level of 5.00.

Factor C (Abrasive Concentration) denotes the concentration of abrasive material used in the process, with a low level of 100.00 and a high level of 200.00.

Factor D (Feed) represents the feed rate of material, with a low level of 30.00 and a high level of 60.00.

Each factor has been defined with specific levels corresponding to the low and high extremes of the experimental range. The standard deviation for each factor is provided as 0.0000, which suggests that these values are fixed and not subject to variation.

**Table 5:** Desirability based optimization table.

Factor	Name	Level	Low level	High level	Std. Dev.	Coding
A	Size	113.74	100.00	140.00	0.0000	Actual
B	SOD	4.99	1.0000	5.00	0.0000	Actual
C	Abr. con.	198.93	100.00	200.00	0.0000	Actual
D	Feed	34.00	30.00	60.00	0.0000	Actual

Two-sided Confidence = 95% Population = 99%.

The prediction Table 6 presents precise estimates for the Mean and Median values of the Response Variable, namely the Material Removal Rate (MRR).

The estimated average Material Removal Rate obtained from the prediction model is 2.88828. The average MRR values expected under specific circumstances or settings of the study’s elements are represented by the mean.

The middle value of the MRR distribution when the data is arranged in ascending order is the predicted median, or 2.91462. The value at which the lower and upper 50% of MRR data are separated is called the median. Statistically speaking, the median is a measure of central tendency that is less affected by outliers or extreme values than the mean. The forecasts are based on statistical models or regression equations created from experimental data. By evaluating the anticipated performance of a system or process under particular circumstances, point-based predictions can assist scholars and practitioners in making well-informed decisions and streamlining their procedures.

**Table 6:** Prediction table for MRR.

Solution	Predicted mean	Predicted median
MRR	2.88828	2.91462

### Model – predictions with AI and ML

The ML algorithms, which are Random Forest, XGBoost and Artificial Neural Network (ANN), are used to predict the Material Removal Rate in the proposed model. The effectiveness of prediction models like Random Forest and XGBoost in predicting MRR, material removal rate in g/min, is evaluated for several criteria. It is done on the training and testing data sets.The mean squared error (MSE), mean absolute error (MAE), and coefficient of determination (R-squared) are commonly employed in AI and ML for model predictions. The efficacy of the different models is shown in Table 7 for comparison.

**Table 7:** Statistics of models.

		*R* ^2^	MSE	MAE
Random forest	Training	0.9974	0.0003	0.0124
	Test	0.9762	0.0016	0.0353
XGBoost	Training	1	0	0.0008
	Test	0.9999	0.0001	0.0065
ANN	Training	1	0	0.0005
	Test	0.9967	0.0013	0.0198

#### Random forest

**Fig. 6:**
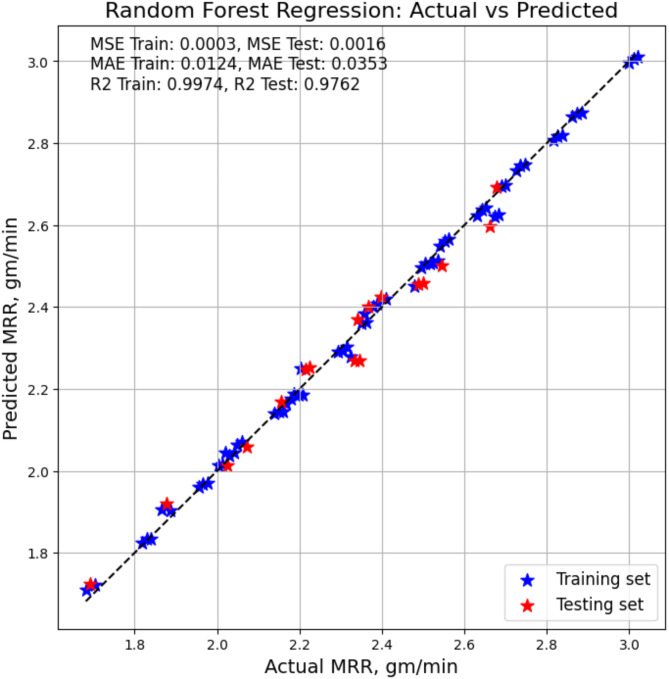
RF model based MRR actual vs. predicted values.

When Random Forest is tested on the training dataset, as indicated in Table 7, its coefficient of determination (R-squared) is 0.9974. This suggests that 99.74% of the variance in response variables can be explained by the model. This high score indicates that the training data and the model fit each other well. Not only that, but the training data’s mean squared error (MSE) is 0.0026, indicating that the average squared difference between the observed and predicted values is not very large. Moreover, the training set’s mean absolute error (MAE) is 0.0091, indicating that the average absolute difference between the predicted and observed values is likewise quite tiny. The Random Forest model achieves a high (R-squared) score of 0.9762 on the testing dataset, indicating its exceptional ability to generalize well to new data. Compared to the training set, the Mean Squared Error (MSE) for the testing data is 0.0238, suggesting a somewhat larger error but still within the range of being deemed relatively low. In a similar vein, the mean absolute error (MAE) for the testing set is 0.0353, which indicates that there is a very small discrepancy between the actual values and the projected values. The Fig. 6 gives the actual vs. predicted MRR based on RF modelling.

#### XGBoost based model – prediction of MRR

**Fig. 7:**
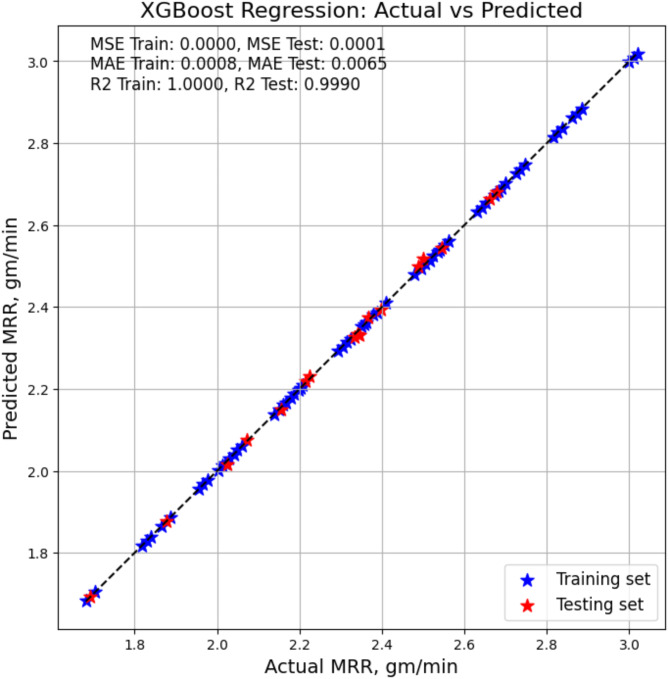
XGBoost model based MRR actual vs. predicted values.

With an R-squared score of 1, the XGBoost model gives an excellent performance on the training dataset, as listed in Table 7. This value indicates that the model is a perfect match and that there are no residual errors. The mean squared error (MSE) and the mean absolute error (MAE) on the training data are both extremely low, coming in at 0.0008 and 0 respectively, indicating that there are very few errors in prediction. A value of 0.9999 for the R-squared statistic indicates that the XGBoost model performs extraordinarily well on the testing dataset. This result indicates that there is low residual error when the model is generalized to fresh data. In a similar vein, the MSE and MAE value on the testing data are remarkably low, coming in at 0.0001 and 0.0065 respectively, which demonstrates the validity and endurance of the model. The Fig. 7 gives the actual vs. predicted values for XGBoost based modelling.

#### ANN – prediction of MRR

**Fig. 8:**
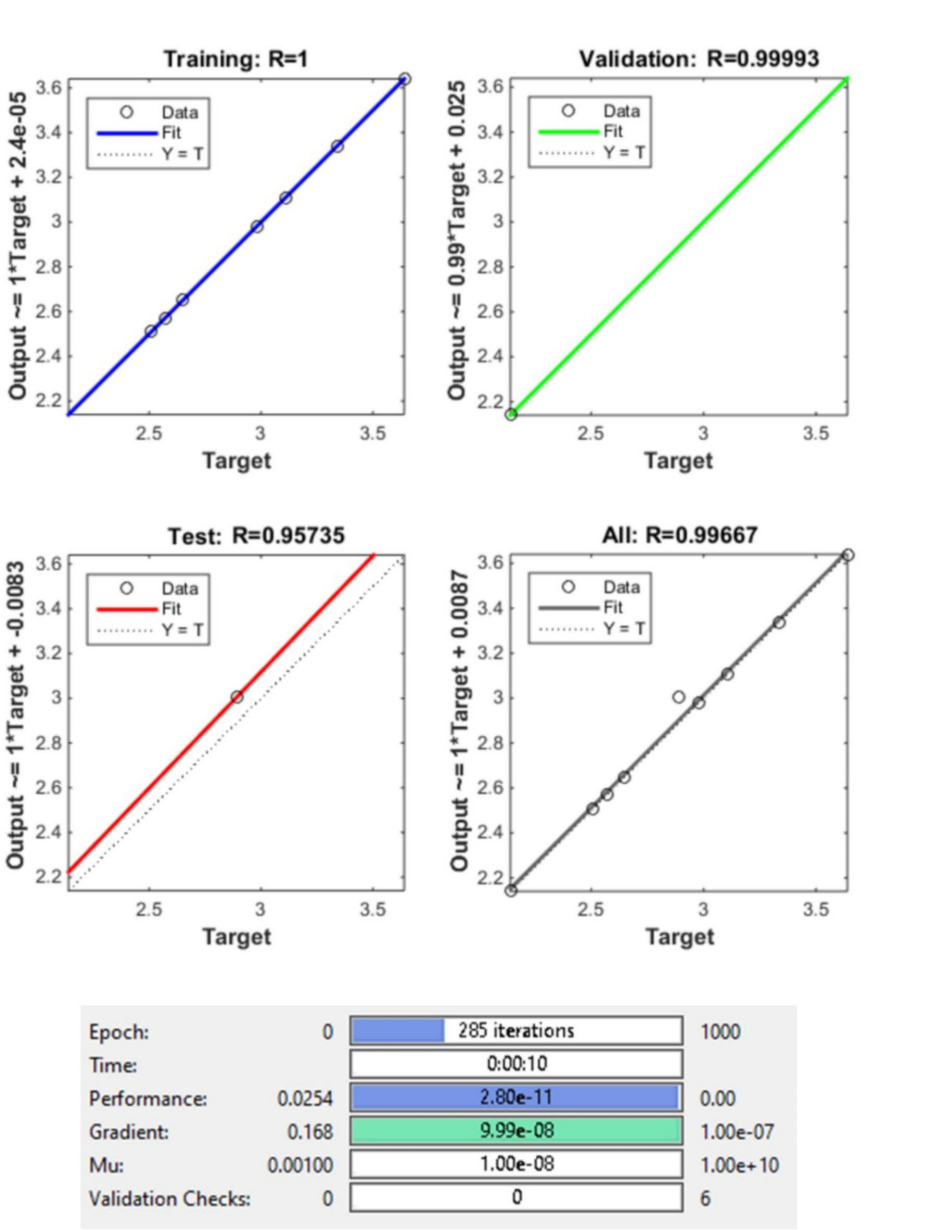
Regression plot for training, validation, testing and overall R value of the model.

**Fig. 9:**
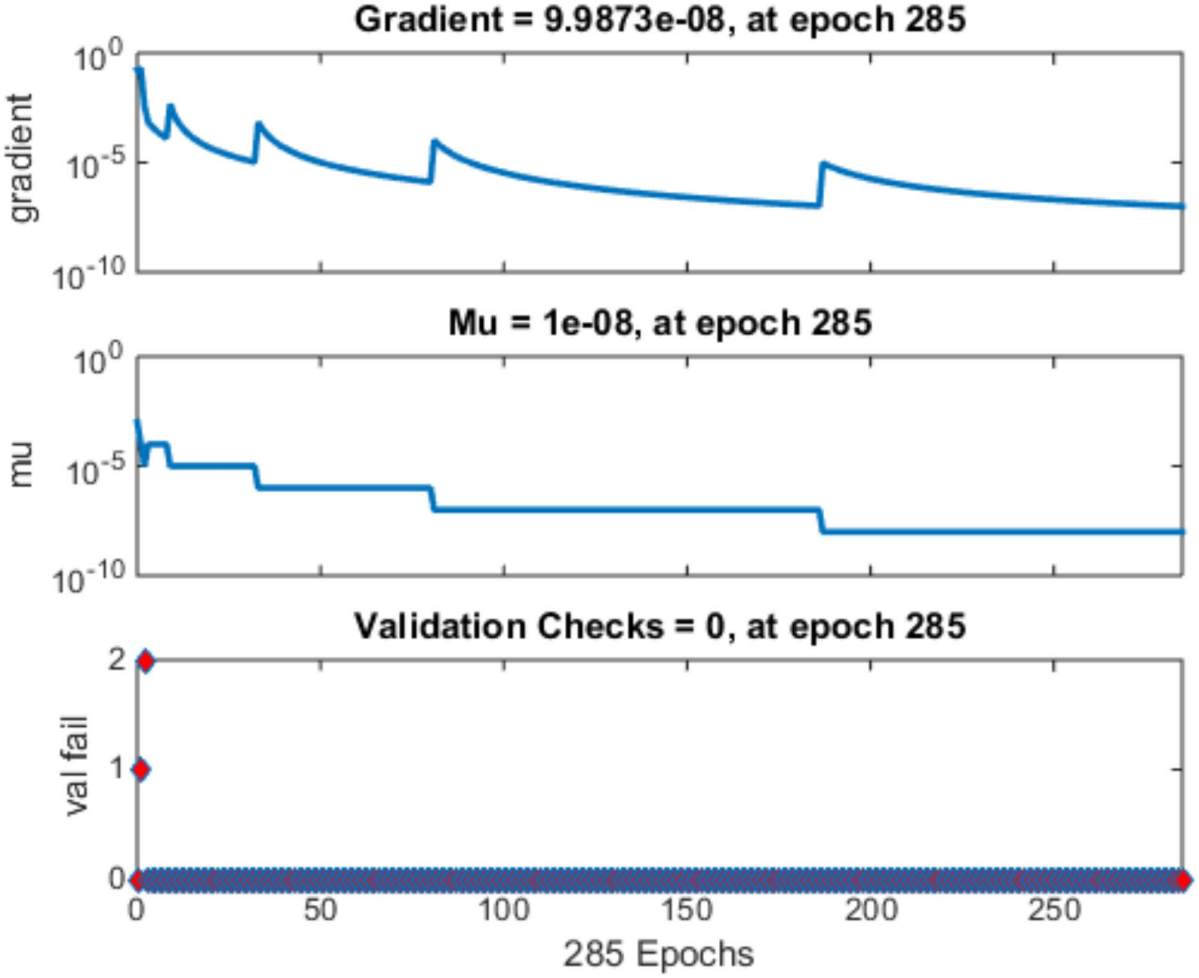
Gradient for validation checks accomplished.

**Fig. 10:**
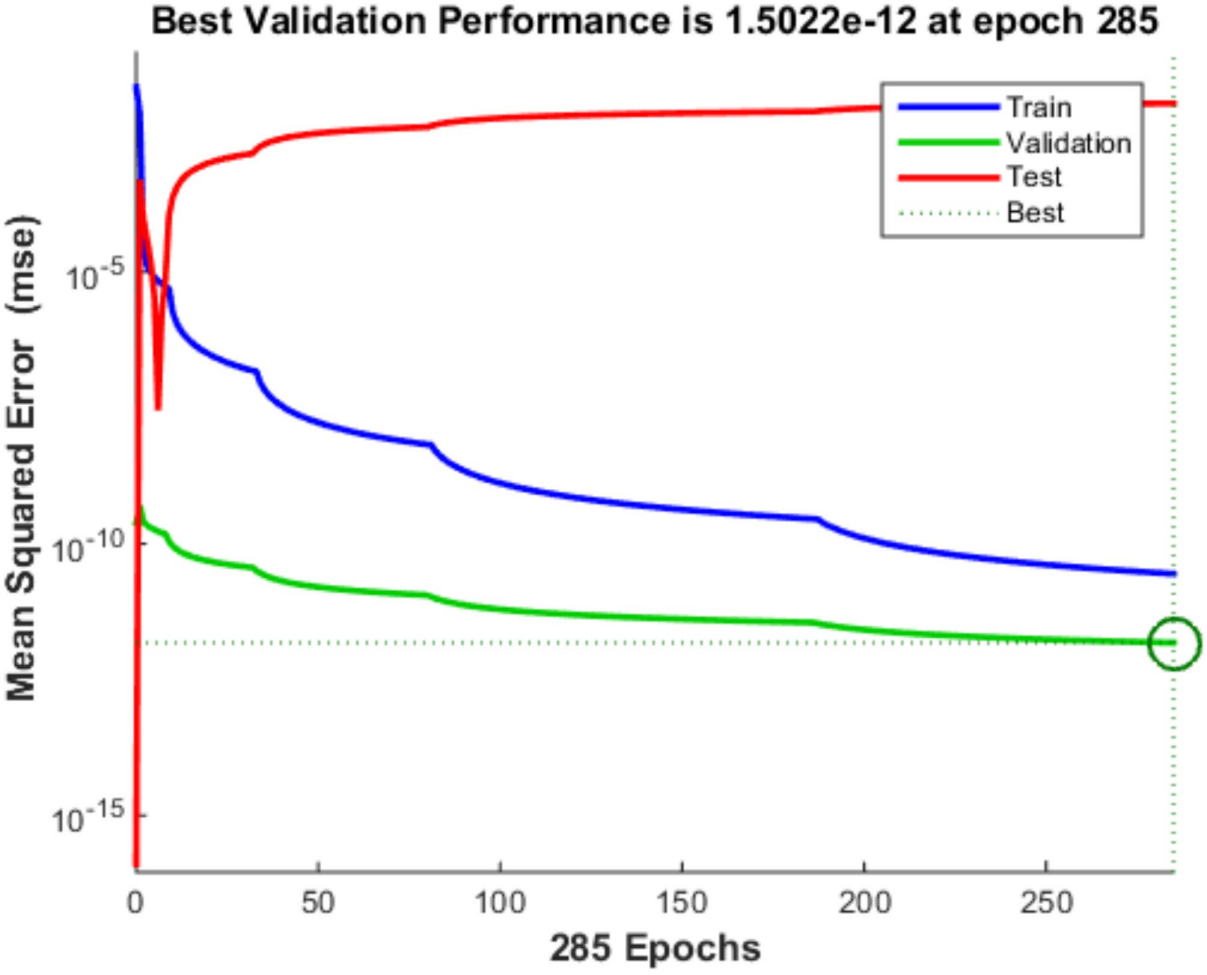
Best validation performance obtained after the 285th iteration.

The ANN model’s performance indicators are assessed. Such facts as an R2 value equal to 1, which says that the network captures 100% of the variance, along with low MSE and MAE values, clearly tell how well an ANN performs its task during the training process. These measurements specify the very exact ability of ANN to identify trends in the train data. In the testing phase, the ANN maintains strong predictive capability, reflected in its high R^2^ value of 0.9967, indicative of its ability to explain nearly 99.67% of the variability in the testing dataset. The relatively low MSE and MAE values during testing further affirm the ANN’s effectiveness in accurately predicting the target variable, suggesting its robust performance and generalizability across unseen data. The Fig. 8 gives the regression plot for training, testing validation and overall R value of the model, while the Fig. 9 gives the gradient of the validation checks accomplished on the ML model, the Fig. 10 gives the best validation performance obtained after the 285th iteration.

The AI and ML models perform well across all assessment criteria on both the training and testing datasets, which indicates that they are useful in predicting the target variable. Thus, the use of AI and ML are helpful in producing accurate predictions. The current work’s outcomes are contrasted with those of Srikanth et al.^28^, who have effectively carried out a number of investigations on optimizations using response surface methods. This required a thorough examination of experimental trials and their results, with an emphasis on important details and validations. The work carried out by Parikh et al.^29^ focused on maximizing experimental factors related to abrasive water jet machining. According to the published data, the optimal values selected not only increased the cutting efficiency of the process, but also optimized the process parameters. In the current work, the MRR increases as the surface roughness decreases. This improvement is attributed to the careful setting of standoff distance and transverse speed during the AWSJ process. MRR was analyzed using RSM, AI, and ML techniques to identify significant factors and optimize the AWSJ process. RSM^30^ developed a quadratic regression model to capture the relationships between input parameters (abrasive size, SOD, concentration, feed rate) and MRR. ANOVA was applied to determine statistically significant factors and interactions, while response surfaces and contour plots visualized these relationships and identified optimal parameter combinations.

AI/ML models^31^, including Random Forest, XGBoost, and ANN, were trained on experimental data with input parameters as predictors and MRR as the target variable. These models, validated through training-testing splits, demonstrated high predictive accuracy, outperforming RSM in capturing non-linear relationships. Metrics such as R^2^, MSE, and MAE confirmed their reliability.

RSM provided statistical insights and parameter interactions, while AI/ML offered precise, data-driven predictions, enabling efficient optimization of MRR with minimal experimentation. Together, these methods provided a comprehensive framework for enhancing AWSJ machining processes.

The findings achieved in this work for cutting CFRP composites using AWSJ are compared with those of Santhanakumar et al.^32^, who used Grey-Based RSM to optimize the parameters for cutting ceramic tiles using abrasive waterjet. In order to preserve quality and productivity in the industrial sector, this study tackles the difficulties in processing ceramic tiles while obtaining accurate cuts and low surface roughness. This has been strongly demonstrated by the research of Uhlmann et al.^33^, which also validates the need of an effective abrasive water jet milling technique for creating hard-to-cut near-net-shaped components. Cutting parameters such jet pressure, abrasive flow rate, standoff distance, and traverse speed were tested experimentally to see how they affected surface roughness and material removal rate. The intricate relationship between process aspects and performance indicators has been effectively reproduced by a number of researchers using Grey Relational RSM approaches^34,35^. The results of the study offer important insights into the ideal parameter configurations for improving the effectiveness and quality of abrasive waterjet machining of ceramic tiles. The Material Removal Rate (MRR) is a critical component of the AWSJ process for machining composites, as demonstrated by methodical testing and study.

The MRR and machining quality of composites can be enhanced by considering factors such as abrasive size, concentration, nozzle standoff distance, and suspension of the composites in water.

## Conclusions

The study compared RSM, ANN, and ML models (XGBoost and Random Forest) for predicting MRR in AWSJ machining. Performance metrics revealed superior predictive accuracy for ML and ANN models over RSM. For RSM, R^2^ was 0.9525, Predicted R^2^ was 0.8208, and MSE was 0.1000. In comparison, ANN achieved R^2^ of 1.0 for training and 0.9967 for testing, with MSE and MAE as low as 0.0013 and 0.0198 in testing. XGBoost outperformed with R^2^ of 1.0 for training and 0.9999 for testing, and MSE and MAE at 0.0001 and 0.0065, respectively. Random Forest also performed well, with R^2^ of 0.9974 for training and 0.9762 for testing.While ML and ANN models excel in predictive accuracy and capturing non-linear relationships, RSM remains valuable for its interpretability. It effectively visualizes parameter interactions (e.g., SOD with other factors) through response surfaces and contour plots, aiding in understanding process dynamics. By integrating the precision of ML/ANN with RSM’s clarity, the study provides a comprehensive framework for optimizing AWSJ machining processes, which are elucidate pointwise as follows.

- The Material Removal Rate (MRR) has been seen to have an impact on the Top Kerf Width (TKW), Back Kerf Width (BKW), and Surface Roughness (SR) while using Abrasive Waterjet Cutting (AWSJ) on CFRP composites.
- The standoff distance (SOD) has a significant effect on the MRR. When employing RSM-based statistical methods for parametric optimization, the effect of SOD together with the abrasive size, abrasive concentration, and feed are taken into consideration.
- RSM readings offer useful insights into the variability of data, but they may not possess the predictive strength and generalization capabilities of ML and ANN models.
- ANN and ML algorithms like XGBoost and Random Forest show good predicting performance with low MSE and MAE and high R^2^ values.
- ML techniques, such as Random Forest and XGBoost, demonstrate remarkable ability to generalize and make accurate predictions, with Artificial Neural Networks (ANN) being a close contender.
- Particularly in composite machining processes such as CFRP, ML and ANN models are highly effective tools for predicting and optimizing machining parameters. They provide accurate insights into Material Removal Rate (MRR), Kerf Width (TKW and BKW), and surface roughness.
- The ML and ANN models exhibit a higher R^2^ value, indicating more prediction compared to the RSM model. However, the RSM model can be used to model and display the interaction of SOD with other factors using standard statistical methods.

The optimized AWSJ process enhances machining efficiency for CFRP composites, achieving higher MRR, reduced surface roughness, and uniform kerf widths while submerged cutting minimizes vibrations and AI/ML reduces experimental effort, benefiting precision industries like aerospace. However, the study is limited to specific parameters and CFRP materials under lab conditions, requiring advanced resources, and may not fully generalize to industrial-scale applications. Future research should explore other materials, broader parameter ranges, real-time control, nozzle wear effects, and hybrid techniques to validate scalability and robustness.

## Data Availability

All data used to support the findings of this study are included within the article.
